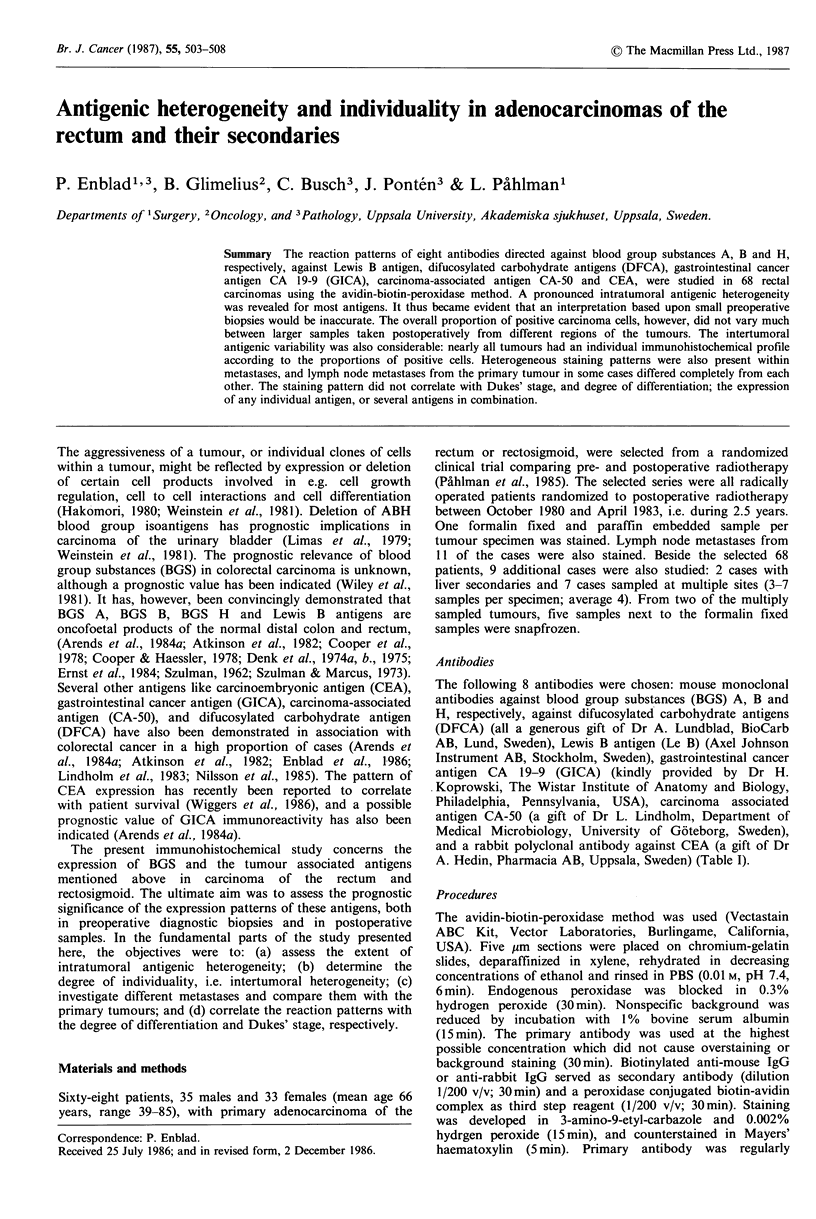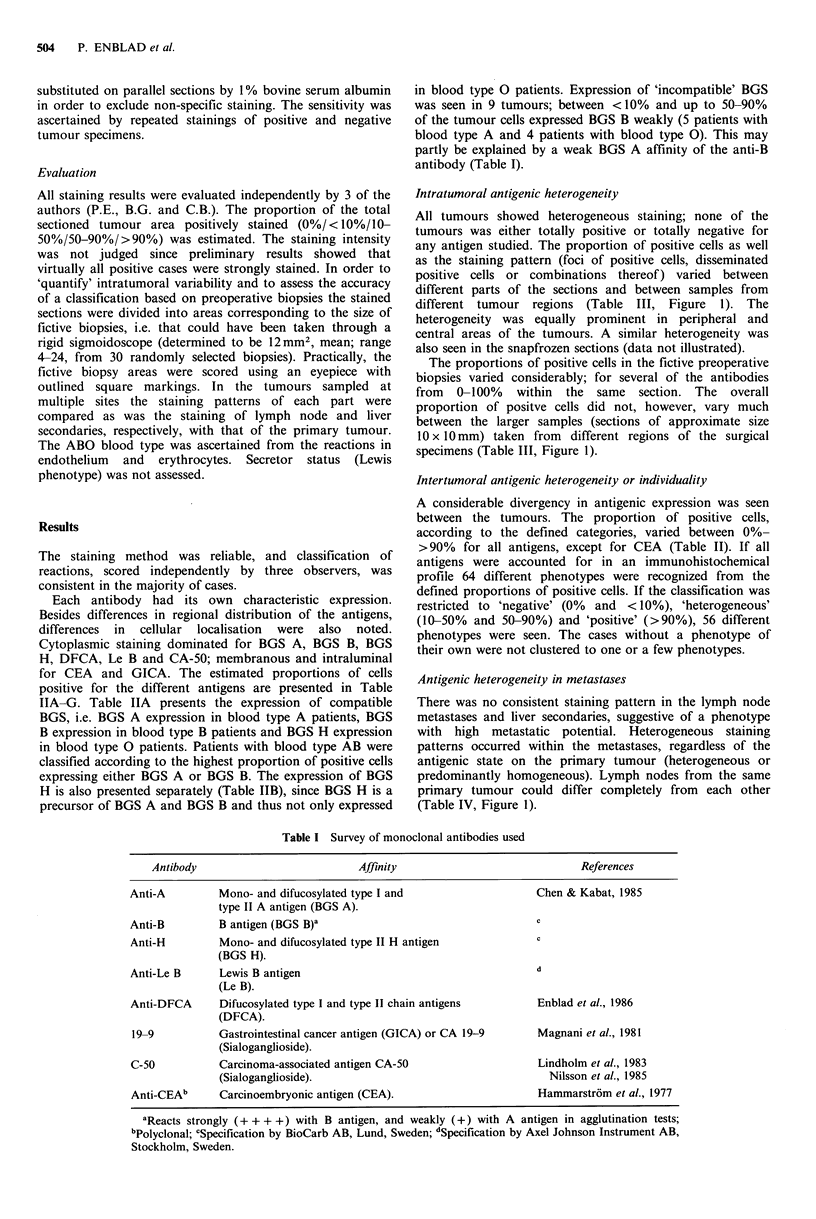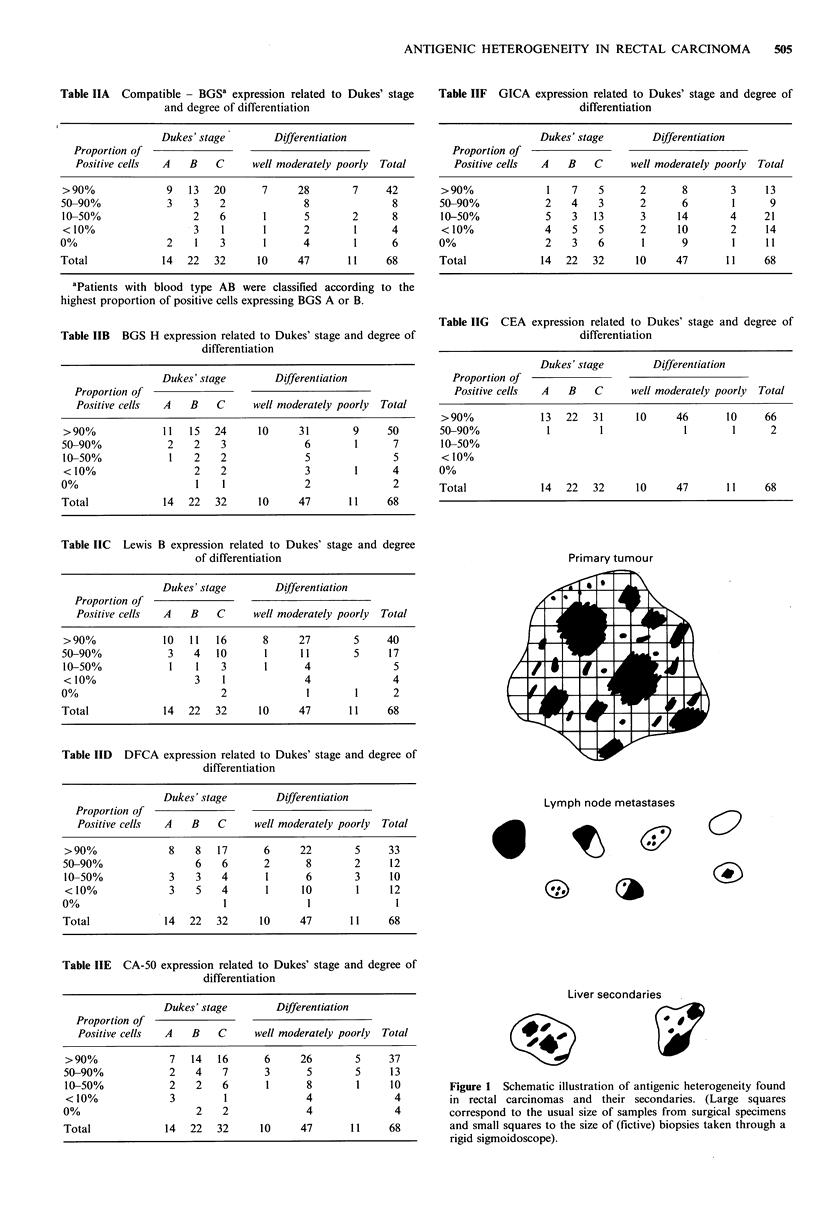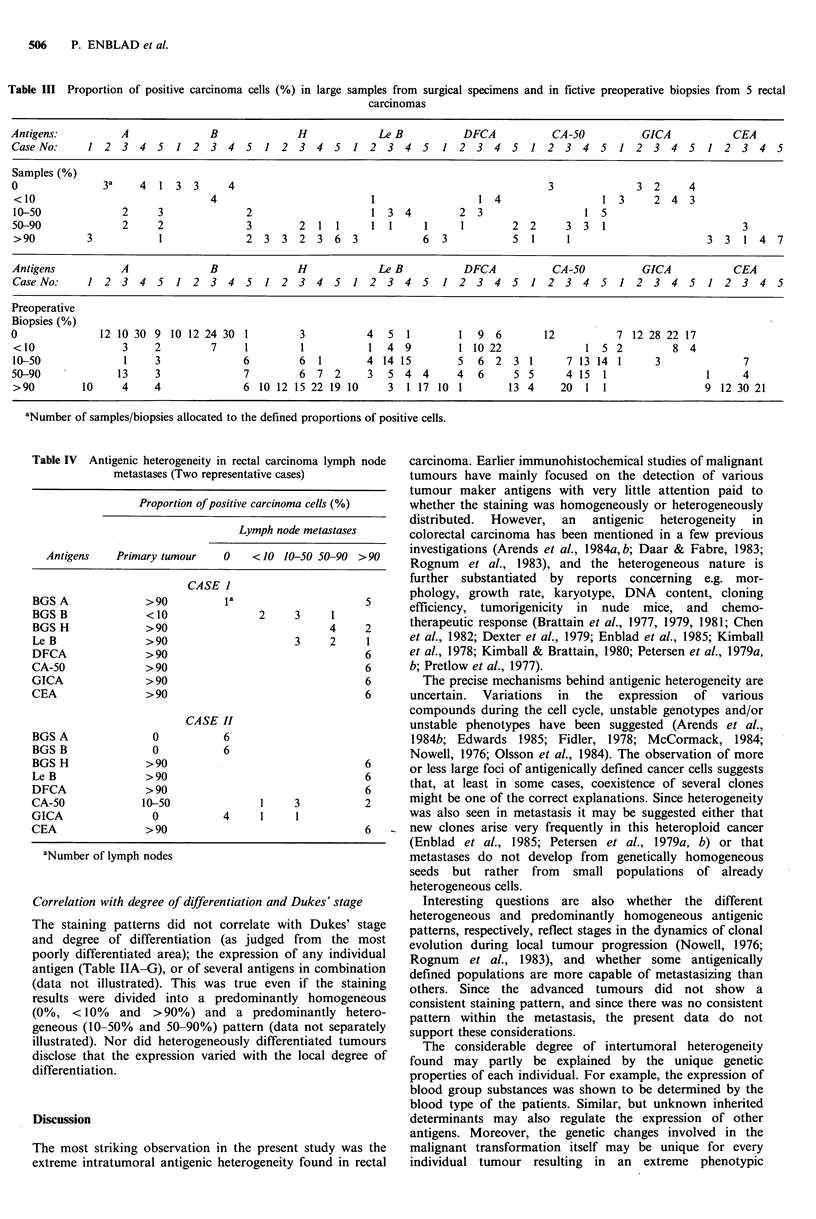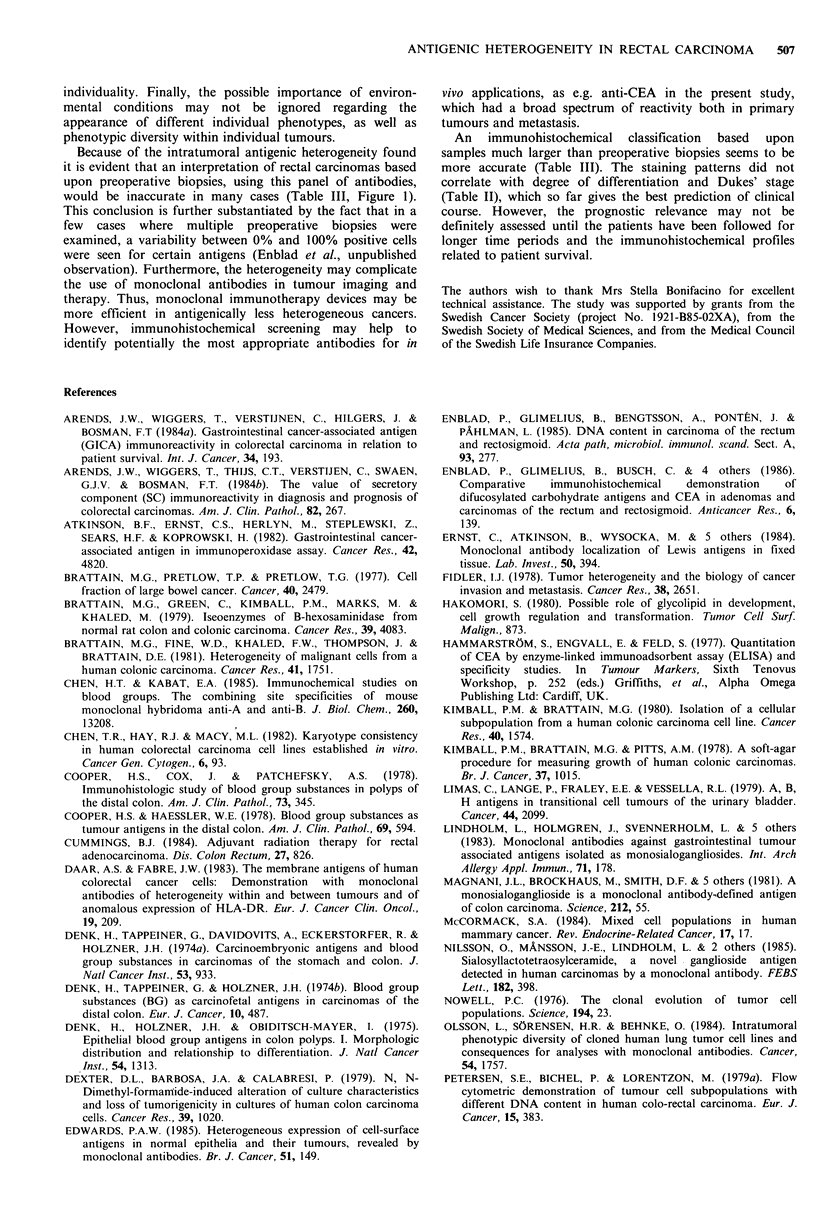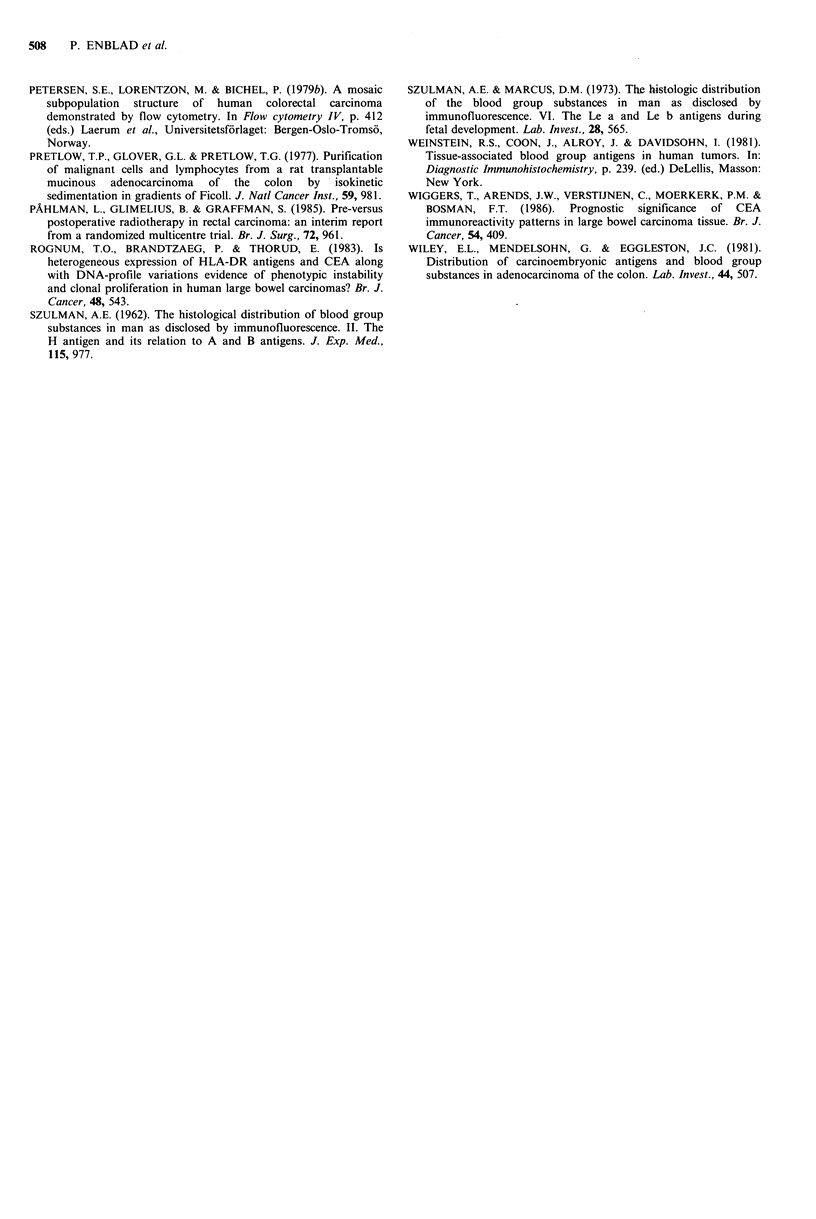# Antigenic heterogeneity and individuality in adenocarcinomas of the rectum and their secondaries.

**DOI:** 10.1038/bjc.1987.102

**Published:** 1987-05

**Authors:** P. Enblad, B. Glimelius, C. Busch, J. Pontén, L. Påhlman

## Abstract

The reaction patterns of eight antibodies directed against blood group substances A, B and H, respectively, against Lewis B antigen, difucosylated carbohydrate antigens (DFCA), gastrointestinal cancer antigen CA 19-9 (GICA), carcinoma-associated antigen CA-50 and CEA, were studied in 68 rectal carcinomas using the avidin-biotin-peroxidase method. A pronounced intratumoral antigenic heterogeneity was revealed for most antigens. It thus became evident that an interpretation based upon small preoperative biopsies would be inaccurate. The overall proportion of positive carcinoma cells, however, did not vary much between larger samples taken postoperatively from different regions of the tumours. The intertumoral antigenic variability was also considerable: nearly all tumours had an individual immunohistochemical profile according to the proportions of positive cells. Heterogeneous staining patterns were also present within metastases, and lymph node metastases from the primary tumour in some cases differed completely from each other. The staining pattern did not correlate with Dukes' stage, and degree of differentiation; the expression of any individual antigen, or several antigens in combination.


*Br. J. Cancer* (1987), **55**, 503–508 © The Macmillan Press Ltd., 1987

### Antigenic heterogeneity and individuality in adenocarcinomas of the rectum and their secondaries

P. Enblad[1,3], B. Glimelius[2], C. Busch[3], J. Pontén[3] & L. Påhlman[1]

*Departments of [1]Surgery, [2]Oncology, and [3]Pathology, Uppsala University, Akademiska sjukhuset, Uppsala, Sweden.*

**Summary** The reaction patterns of eight antibodies directed against blood group substances A, B and H, respectively, against Lewis B antigen, difucosylated carbohydrate antigens (DFCA), gastrointestinal cancer antigen CA 19-9 (GICA), carcinoma-associated antigen CA-50 and CEA, were studied in 68 rectal carcinomas using the avidin-biotin-peroxidase method. A pronounced intratumoral antigenic heterogeneity was revealed for most antigens. It thus became evident that an interpretation based upon small preoperative biopsies would be inaccurate. The overall proportion of positive carcinoma cells, however, did not vary much between larger samples taken postoperatively from different regions of the tumours. The intertumoral antigenic variability was also considerable: nearly all tumours had an individual immunohistochemical profile according to the proportions of positive cells. Heterogeneous staining patterns were also present within metastases, and lymph node metastases from the primary tumour in some cases differed completely from each other. The staining pattern did not correlate with Dukes' stage, and degree of differentiation; the expression of any individual antigen, or several antigens in combination.

The aggressiveness of a tumour, or individual clones of cells within a tumour, might be reflected by expression or deletion of certain cell products involved in e.g. cell growth regulation, cell to cell interactions and cell differentiation (Hakomori, 1980; Weinstein *et al.*, 1981). Deletion of ABH blood group isoantigens has prognostic implications in carcinoma of the urinary bladder (Limas *et al.*, 1979; Weinstein *et al.*, 1981). The prognostic relevance of blood group substances (BGS) in colorectal carcinoma is unknown, although a prognostic value has been indicated (Wiley *et al.*, 1981). It has, however, been convincingly demonstrated that BGS A, BGS B, BGS H and Lewis B antigens are oncofoetal products of the normal distal colon and rectum, (Arends *et al.*, 1984*a*; Atkinson *et al.*, 1982; Cooper *et al.*, 1978; Cooper & Haessler, 1978; Denk *et al.*, 1974*a*, *b*., 1975; Ernst *et al.*, 1984; Szulman, 1962; Szulman & Marcus, 1973). Several other antigens like carcinoembryonic antigen (CEA), gastrointestinal cancer antigen (GICA), carcinoma-associated antigen (CA-50), and difucosylated carbohydrate antigen (DFCA) have also been demonstrated in association with colorectal cancer in a high proportion of cases (Arends *et al.*, 1984*a*; Atkinson *et al.*, 1982; Enblad *et al.*, 1986; Lindholm *et al.*, 1983; Nilsson *et al.*, 1985). The pattern of CEA expression has recently been reported to correlate with patient survival (Wiggers *et al.*, 1986), and a possible prognostic value of GICA immunoreactivity has also been indicated (Arends *et al.*, 1984*a*).

The present immunohistochemical study concerns the expression of BGS and the tumour associated antigens mentioned above in carcinoma of the rectum and rectosigmoid. The ultimate aim was to assess the prognostic significance of the expression patterns of these antigens, both in preoperative diagnostic biopsies and in postoperative samples. In the fundamental parts of the study presented here, the objectives were to: (a) assess the extent of intratumoral antigenic heterogeneity; (b) determine the degree of individuality, i.e. intertumoral heterogeneity; (c) investigate different metastases and compare them with the primary tumours; and (d) correlate the reaction patterns with the degree of differentiation and Dukes' stage, respectively.

### Materials and methods

Sixty-eight patients, 35 males and 33 females (mean age 66 years, range 39–85), with primary adenocarcinoma of the rectum or rectosigmoid, were selected from a randomized clinical trial comparing pre- and postoperative radiotherapy (Påhlman *et al.*, 1985). The selected series were all radically operated patients randomized to postoperative radiotherapy between October 1980 and April 1983, i.e. during 2.5 years. One formalin fixed and paraffin embedded sample per tumour specimen was stained. Lymph node metastases from 11 of the cases were also stained. Beside the selected 68 patients, 9 additional cases were also studied: 2 cases with liver secondaries and 7 cases sampled at multiple sites (3–7 samples per specimen; average 4). From two of the multiply sampled tumours, five samples next to the formalin fixed samples were snapfrozen.

### *Antibodies*

The following 8 antibodies were chosen: mouse monoclonal antibodies against blood group substances (BGS) A, B and H, respectively, against difucosylated carbohydrate antigens (DFCA) (all a generous gift of Dr A. Lundblad, BioCarb AB, Lund, Sweden), Lewis B antigen (Le B) (Axel Johnson Instrument AB, Stockholm, Sweden), gastrointestinal cancer antigen CA 19–9 (GICA) (kindly provided by Dr H. Koprowski, The Wistar Institute of Anatomy and Biology, Philadelphia, Pennsylvania, USA), carcinoma associated antigen CA-50 (a gift of Dr L. Lindholm, Department of Medical Microbiology, University of Göteborg, Sweden), and a rabbit polyclonal antibody against CEA (a gift of Dr A. Hedin, Pharmacia AB, Uppsala, Sweden) (Table I).

### *Procedures*

The avidin-biotin-peroxidase method was used (Vectastain ABC Kit, Vector Laboratories, Burlingame, California, USA). Five $\mu$m sections were placed on chromium-gelatin slides, deparaffinized in xylene, rehydrated in decreasing concentrations of ethanol and rinsed in PBS (0.01 M, pH 7.4, 6 min). Endogenous peroxidase was blocked in 0.3% hydrogen peroxide (30 min). Nonspecific background was reduced by incubation with 1% bovine serum albumin (15 min). The primary antibody was used at the highest possible concentration which did not cause overstaining or background staining (30 min). Biotinylated anti-mouse IgG or anti-rabbit IgG served as secondary antibody (dilution 1/200 v/v; 30 min) and a peroxidase conjugated biotin-avidin complex as third step reagent (1/200 v/v; 30 min). Staining was developed in 3-amino-9-etyl-carbazole and 0.002% hydrgen peroxide (15 min), and counterstained in Mayers' haematoxylin (5 min). Primary antibody was regularly

Correspondence: P. Enblad.
Received 25 July 1986; and in revised form, 2 December 1986.

substituted on parallel sections by 1% bovine serum albumin in order to exclude non-specific staining. The sensitivity was ascertained by repeated stainings of positive and negative tumour specimens.

*Evaluation*

All staining results were evaluated independently by 3 of the authors (P.E., B.G. and C.B.). The proportion of the total sectioned tumour area positively stained (0%/<10%/10–50%/50–90%/>90%) was estimated. The staining intensity was not judged since preliminary results showed that virtually all positive cases were strongly stained. In order to 'quantify' intratumoral variability and to assess the accuracy of a classification based on preoperative biopsies the stained sections were divided into areas corresponding to the size of fictive biopsies, i.e. that could have been taken through a rigid sigmoidoscope (determined to be 12 mm$^2$, mean; range 4–24, from 30 randomly selected biopsies). Practically, the fictive biopsy areas were scored using an eyepiece with outlined square markings. In the tumours sampled at multiple sites the staining patterns of each part were compared as was the staining of lymph node and liver secondaries, respectively, with that of the primary tumour. The ABO blood type was ascertained from the reactions in endothelium and erythrocytes. Secretor status (Lewis phenotype) was not assessed.

### Results

The staining method was reliable, and classification of reactions, scored independently by three observers, was consistent in the majority of cases.

Each antibody had its own characteristic expression. Besides differences in regional distribution of the antigens, differences in cellular localisation were also noted. Cytoplasmic staining dominated for BGS A, BGS B, BGS H, DFCA, Le B and CA-50; membranous and intraluminal for CEA and GICA. The estimated proportions of cells positive for the different antigens are presented in Table IIA–G. Table IIA presents the expression of compatible BGS, i.e. BGS A expression in blood type A patients, BGS B expression in blood type B patients and BGS H expression in blood type O patients. Patients with blood type AB were classified according to the highest proportion of positive cells expressing either BGS A or BGS B. The expression of BGS H is also presented separately (Table IIB), since BGS H is a precursor of BGS A and BGS B and thus not only expressed in blood type O patients. Expression of 'incompatible' BGS was seen in 9 tumours; between <10% and up to 50–90% of the tumour cells expressed BGS B weakly (5 patients with blood type A and 4 patients with blood type O). This may partly be explained by a weak BGS A affinity of the anti-B antibody (Table I).

*Intratumoral antigenic heterogeneity*

All tumours showed heterogeneous staining; none of the tumours was either totally positive or totally negative for any antigen studied. The proportion of positive cells as well as the staining pattern (foci of positive cells, disseminated positive cells or combinations thereof) varied between different parts of the sections and between samples from different tumour regions (Table III, Figure 1). The heterogeneity was equally prominent in peripheral and central areas of the tumours. A similar heterogeneity was also seen in the snapfrozen sections (data not illustrated).

The proportions of positive cells in the fictive preoperative biopsies varied considerably; for several of the antibodies from 0–100% within the same section. The overall proportion of positve cells did not, however, vary much between the larger samples (sections of approximate size 10 × 10 mm) taken from different regions of the surgical specimens (Table III, Figure 1).

*Intertumoral antigenic heterogeneity or individuality*

A considerable divergency in antigenic expression was seen between the tumours. The proportion of positive cells, according to the defined categories, varied between 0%–>90% for all antigens, except for CEA (Table II). If all antigens were accounted for in an immunohistochemical profile 64 different phenotypes were recognized from the defined proportions of positive cells. If the classification was restricted to 'negative' (0% and <10%), 'heterogeneous' (10–50% and 50–90%) and 'positive' (>90%), 56 different phenotypes were seen. The cases without a phenotype of their own were not clustered to one or a few phenotypes.

*Antigenic heterogeneity in metastases*

There was no consistent staining pattern in the lymph node metastases and liver secondaries, suggestive of a phenotype with high metastatic potential. Heterogeneous staining patterns occurred within the metastases, regardless of the antigenic state on the primary tumour (heterogeneous or predominantly homogeneous). Lymph nodes from the same primary tumour could differ completely from each other (Table IV, Figure 1).

**Table I** Survey of monoclonal antibodies used

| *Antibody* | *Affinity* | *References* |
|---|---|---|
| Anti-A | Mono- and difucosylated type I and type II A antigen (BGS A). | Chen & Kabat, 1985 |
| Anti-B | B antigen (BGS B)[a] | [c] |
| Anti-H | Mono- and difucosylated type II H antigen (BGS H). | [c] |
| Anti-Le B | Lewis B antigen (Le B). | [d] |
| Anti-DFCA | Difucosylated type I and type II chain antigens (DFCA). | Enblad *et al.*, 1986 |
| 19–9 | Gastrointestinal cancer antigen (GICA) or CA 19–9 (Sialoganglioside). | Magnani *et al.*, 1981 |
| C-50 | Carcinoma-associated antigen CA-50 (Sialoganglioside). | Lindholm *et al.*, 1983<br>Nilsson *et al.*, 1985 |
| Anti-CEA[b] | Carcinoembryonic antigen (CEA). | Hammarström *et al.*, 1977 |

[a]Reacts strongly (++++) with B antigen, and weakly (+) with A antigen in agglutination tests; [b]Polyclonal; [c]Specification by BioCarb AB, Lund, Sweden; [d]Specification by Axel Johnson Instrument AB, Stockholm, Sweden.

**Table IIA** Compatible – BGS[a] expression related to Dukes' stage and degree of differentiation

| *Proportion of Positive cells* | *Dukes' stage* | | | *Differentiation* | | | |
|---|---|---|---|---|---|---|---|
| | *A* | *B* | *C* | *well* | *moderately* | *poorly* | *Total* |
| >90% | 9 | 13 | 20 | 7 | 28 | 7 | 42 |
| 50–90% | 3 | 3 | 2 | | 8 | | 8 |
| 10–50% | | 2 | 6 | 1 | 5 | 2 | 8 |
| <10% | | 3 | 1 | 1 | 2 | 1 | 4 |
| 0% | 2 | 1 | 3 | 1 | 4 | 1 | 6 |
| Total | 14 | 22 | 32 | 10 | 47 | 11 | 68 |

[a]Patients with blood type AB were classified according to the highest proportion of positive cells expressing BGS A or B.

**Table IIB** BGS H expression related to Dukes' stage and degree of differentiation

| *Proportion of Positive cells* | *Dukes' stage* | | | *Differentiation* | | | |
|---|---|---|---|---|---|---|---|
| | *A* | *B* | *C* | *well* | *moderately* | *poorly* | *Total* |
| >90% | 11 | 15 | 24 | 10 | 31 | 9 | 50 |
| 50–90% | 2 | 2 | 3 | | 6 | 1 | 7 |
| 10–50% | 1 | 2 | 2 | | 5 | | 5 |
| <10% | | 2 | 2 | | 3 | 1 | 4 |
| 0% | | 1 | 1 | | 2 | | 2 |
| Total | 14 | 22 | 32 | 10 | 47 | 11 | 68 |

**Table IIC** Lewis B expression related to Dukes' stage and degree of differentiation

| *Proportion of Positive cells* | *Dukes' stage* | | | *Differentiation* | | | |
|---|---|---|---|---|---|---|---|
| | *A* | *B* | *C* | *well* | *moderately* | *poorly* | *Total* |
| >90% | 10 | 11 | 16 | 8 | 27 | 5 | 40 |
| 50–90% | 3 | 4 | 10 | 1 | 11 | 5 | 17 |
| 10–50% | 1 | 1 | 3 | 1 | 4 | | 5 |
| <10% | | 3 | 1 | | 4 | | 4 |
| 0% | | | 2 | | 1 | 1 | 2 |
| Total | 14 | 22 | 32 | 10 | 47 | 11 | 68 |

**Table IID** DFCA expression related to Dukes' stage and degree of differentiation

| *Proportion of Positive cells* | *Dukes' stage* | | | *Differentiation* | | | |
|---|---|---|---|---|---|---|---|
| | *A* | *B* | *C* | *well* | *moderately* | *poorly* | *Total* |
| >90% | 8 | 8 | 17 | 6 | 22 | 5 | 33 |
| 50–90% | | 6 | 6 | 2 | 8 | 2 | 12 |
| 10–50% | 3 | 3 | 4 | 1 | 6 | 3 | 10 |
| <10% | 3 | 5 | 4 | 1 | 10 | 1 | 12 |
| 0% | | | 1 | | 1 | | 1 |
| Total | 14 | 22 | 32 | 10 | 47 | 11 | 68 |

**Table IIE** CA-50 expression related to Dukes' stage and degree of differentiation

| *Proportion of Positive cells* | *Dukes' stage* | | | *Differentiation* | | | |
|---|---|---|---|---|---|---|---|
| | *A* | *B* | *C* | *well* | *moderately* | *poorly* | *Total* |
| >90% | 7 | 14 | 16 | 6 | 26 | 5 | 37 |
| 50–90% | 2 | 4 | 7 | 3 | 5 | 5 | 13 |
| 10–50% | 2 | 2 | 6 | 1 | 8 | 1 | 10 |
| <10% | 3 | | 1 | | 4 | | 4 |
| 0% | | 2 | 2 | | 4 | | 4 |
| Total | 14 | 22 | 32 | 10 | 47 | 11 | 68 |

**Table IIF** GICA expression related to Dukes' stage and degree of differentiation

| *Proportion of Positive cells* | *Dukes' stage* | | | *Differentiation* | | | |
|---|---|---|---|---|---|---|---|
| | *A* | *B* | *C* | *well* | *moderately* | *poorly* | *Total* |
| >90% | 1 | 7 | 5 | 2 | 8 | 3 | 13 |
| 50–90% | 2 | 4 | 3 | 2 | 6 | 1 | 9 |
| 10–50% | 5 | 3 | 13 | 3 | 14 | 4 | 21 |
| <10% | 4 | 5 | 5 | 2 | 10 | 2 | 14 |
| 0% | 2 | 3 | 6 | 1 | 9 | 1 | 11 |
| Total | 14 | 22 | 32 | 10 | 47 | 11 | 68 |

**Table IIG** CEA expression related to Dukes' stage and degree of differentiation

| *Proportion of Positive cells* | *Dukes' stage* | | | *Differentiation* | | | |
|---|---|---|---|---|---|---|---|
| | *A* | *B* | *C* | *well* | *moderately* | *poorly* | *Total* |
| >90% | 13 | 22 | 31 | 10 | 46 | 10 | 66 |
| 50–90% | 1 | | 1 | | 1 | 1 | 2 |
| 10–50% | | | | | | | |
| <10% | | | | | | | |
| 0% | | | | | | | |
| Total | 14 | 22 | 32 | 10 | 47 | 11 | 68 |

**Figure 1** Schematic illustration of antigenic heterogeneity found in rectal carcinomas and their secondaries. (Large squares correspond to the usual size of samples from surgical specimens and small squares to the size of (fictive) biopsies taken through a rigid sigmoidoscope).

**Table III** Proportion of positive carcinoma cells (%) in large samples from surgical specimens and in fictive preoperative biopsies from 5 rectal carcinomas

| *Antigens:* | *A* | | | | | *B* | | | | | *H* | | | | | *Le B* | | | | |
|---|---|---|---|---|---|---|---|---|---|---|---|---|---|---|---|---|---|---|---|---|
| *Case No:* | *1* | *2* | *3* | *4* | *5* | *1* | *2* | *3* | *4* | *5* | *1* | *2* | *3* | *4* | *5* | *1* | *2* | *3* | *4* | *5* |
| Samples (%) | | | | | | | | | | | | | | | | | | | | |
| 0 | | 3[a] | | 4 | 1 | 3 | 3 | | 4 | | | | | | | | | | | |
| <10 | | | | | | | | 4 | | | | | | | | | 1 | | | |
| 10–50 | | | 2 | | 3 | | | | | 2 | | | | | | | 1 | 3 | 4 | |
| 50–90 | | | 2 | | 2 | | | | | 3 | | | 2 | 1 | 1 | | 1 | 1 | | 1 |
| >90 | 3 | | | | 1 | | | | | 2 | 3 | 3 | 2 | 3 | 6 | 3 | | | | 6 |
| *Antigens* | *A* | | | | | *B* | | | | | *H* | | | | | *Le B* | | | | |
| *Case No:* | *1* | *2* | *3* | *4* | *5* | *1* | *2* | *3* | *4* | *5* | *1* | *2* | *3* | *4* | *5* | *1* | *2* | *3* | *4* | *5* |
| Preoperative Biopsies (%) | | | | | | | | | | | | | | | | | | | | |
| 0 | | 12 | 10 | 30 | 9 | 10 | 12 | 24 | 30 | 1 | | | 3 | | | | 4 | 5 | 1 | |
| <10 | | | 3 | | 2 | | | 7 | | 1 | | | 1 | | | | 1 | 4 | 9 | |
| 10–50 | | | 1 | | 3 | | | | | 6 | | | 6 | 1 | | | 4 | 14 | 15 | |
| 50–90 | | | 13 | | 3 | | | | | 7 | | | 6 | 7 | 2 | | 3 | 5 | 4 | 4 |
| >90 | 10 | | 4 | | 4 | | | | | 6 | 10 | 12 | 15 | 22 | 19 | 10 | | 3 | 1 | 17 |

| *Antigens:* | *DFCA* | | | | | *CA-50* | | | | | *GICA* | | | | | *CEA* | | | | |
|---|---|---|---|---|---|---|---|---|---|---|---|---|---|---|---|---|---|---|---|---|
| *Case No:* | *1* | *2* | *3* | *4* | *5* | *1* | *2* | *3* | *4* | *5* | *1* | *2* | *3* | *4* | *5* | *1* | *2* | *3* | *4* | *5* |
| Samples (%) | | | | | | | | | | | | | | | | | | | | |
| 0 | | | | | | | 3 | | | | | 3 | 2 | | 4 | | | | | |
| <10 | | | 1 | 4 | | | | | | 1 | 3 | | 2 | 4 | 3 | | | | | |
| 10–50 | | 2 | 3 | | | | | | 1 | 5 | | | | | | | | | | |
| 50–90 | | 1 | | | 2 | 2 | | 3 | 3 | 1 | | | | | | | | 3 | | |
| >90 | 3 | | | | 5 | 1 | | 1 | | | | | | | | 3 | 3 | 1 | 4 | 7 |
| *Antigens* | *DFCA* | | | | | *CA-50* | | | | | *GICA* | | | | | *CEA* | | | | |
| *Case No:* | *1* | *2* | *3* | *4* | *5* | *1* | *2* | *3* | *4* | *5* | *1* | *2* | *3* | *4* | *5* | *1* | *2* | *3* | *4* | *5* |
| Preoperative Biopsies (%) | | | | | | | | | | | | | | | | | | | | |
| 0 | | 1 | 9 | 6 | | | 12 | | | | 7 | 12 | 28 | 22 | 17 | | | | | |
| <10 | | 1 | 10 | 22 | | | | | 1 | 5 | 2 | | | 8 | 4 | | | | | |
| 10–50 | | 5 | 6 | 2 | 3 | 1 | | 7 | 13 | 14 | 1 | | 3 | | | | | 7 | | |
| 50–90 | | 4 | 6 | | 5 | 5 | | 4 | 15 | 1 | | | | | | 1 | | 4 | | |
| >90 | 10 | 1 | | | 13 | 4 | | 20 | 1 | 1 | | | | | | 9 | 12 | 30 | 21 | |

[a]Number of samples/biopsies allocated to the defined proportions of positive cells.

**Table IV** Antigenic heterogeneity in rectal carcinoma lymph node metastases (Two representative cases)

| | *Proportion of positive carcinoma cells* (%) | | | | | |
|---|---|---|---|---|---|---|
| | | *Lymph node metastases* | | | | |
| *Antigens* | *Primary tumour* | *0* | *<10* | *10–50* | *50–90* | *>90* |
| | | *CASE I* | | | | |
| BGS A | >90 | 1[a] | | | | 5 |
| BGS B | <10 | | 2 | 3 | 1 | |
| BGS H | >90 | | | | 4 | 2 |
| Le B | >90 | | | 3 | 2 | 1 |
| DFCA | >90 | | | | | 6 |
| CA-50 | >90 | | | | | 6 |
| GICA | >90 | | | | | 6 |
| CEA | >90 | | | | | 6 |
| | | *CASE II* | | | | |
| BGS A | 0 | 6 | | | | |
| BGS B | 0 | 6 | | | | |
| BGS H | >90 | | | | | 6 |
| Le B | >90 | | | | | 6 |
| DFCA | >90 | | | | | 6 |
| CA-50 | 10–50 | | 1 | 3 | | 2 |
| GICA | 0 | 4 | 1 | 1 | | |
| CEA | >90 | | | | | 6 |

[a]Number of lymph nodes

*Correlation with degree of differentiation and Dukes' stage*

The staining patterns did not correlate with Dukes' stage and degree of differentiation (as judged from the most poorly differentiated area); the expression of any individual antigen (Table IIA–G), or of several antigens in combination (data not illustrated). This was true even if the staining results were divided into a predominantly homogeneous (0%, <10% and >90%) and a predominantly heterogeneous (10–50% and 50–90%) pattern (data not separately illustrated). Nor did heterogeneously differentiated tumours disclose that the expression varied with the local degree of differentiation.

### Discussion

The most striking observation in the present study was the extreme intratumoral antigenic heterogeneity found in rectal carcinoma. Earlier immunohistochemical studies of malignant tumours have mainly focused on the detection of various tumour maker antigens with very little attention paid to whether the staining was homogeneously or heterogeneously distributed. However, an antigenic heterogeneity in colorectal carcinoma has been mentioned in a few previous investigations (Arends *et al.*, 1984*a*, *b*; Daar & Fabre, 1983; Rognum *et al.*, 1983), and the heterogeneous nature is further substantiated by reports concerning e.g. morphology, growth rate, karyotype, DNA content, cloning efficiency, tumorigenicity in nude mice, and chemotherapeutic response (Brattain *et al.*, 1977, 1979, 1981; Chen *et al.*, 1982; Dexter *et al.*, 1979; Enblad *et al.*, 1985; Kimball *et al.*, 1978; Kimball & Brattain, 1980; Petersen *et al.*, 1979*a*, *b*; Pretlow *et al.*, 1977).

The precise mechanisms behind antigenic heterogeneity are uncertain. Variations in the expression of various compounds during the cell cycle, unstable genotypes and/or unstable phenotypes have been suggested (Arends *et al.*, 1984*b*; Edwards 1985; Fidler, 1978; McCormack, 1984; Nowell, 1976; Olsson *et al.*, 1984). The observation of more or less large foci of antigenically defined cancer cells suggests that, at least in some cases, coexistence of several clones might be one of the correct explanations. Since heterogeneity was also seen in metastasis it may be suggested either that new clones arise very frequently in this heteroploid cancer (Enblad *et al.*, 1985; Petersen *et al.*, 1979*a*, *b*) or that metastases do not develop from genetically homogeneous seeds but rather from small populations of already heterogeneous cells.

Interesting questions are also whether the different heterogeneous and predominantly homogeneous antigenic patterns, respectively, reflect stages in the dynamics of clonal evolution during local tumour progression (Nowell, 1976; Rognum *et al.*, 1983), and whether some antigenically defined populations are more capable of metastasizing than others. Since the advanced tumours did not show a consistent staining pattern, and since there was no consistent pattern within the metastasis, the present data do not support these considerations.

The considerable degree of intertumoral heterogeneity found may partly be explained by the unique genetic properties of each individual. For example, the expression of blood group substances was shown to be determined by the blood type of the patients. Similar, but unknown inherited determinants may also regulate the expression of other antigens. Moreover, the genetic changes involved in the malignant transformation itself may be unique for every individual tumour resulting in an extreme phenotypic individuality. Finally, the possible importance of environmental conditions may not be ignored regarding the appearance of different individual phenotypes, as well as phenotypic diversity within individual tumours.

Because of the intratumoral antigenic heterogeneity found it is evident that an interpretation of rectal carcinomas based upon preoperative biopsies, using this panel of antibodies, would be inaccurate in many cases (Table III, Figure 1). This conclusion is further substantiated by the fact that in a few cases where multiple preoperative biopsies were examined, a variability between 0% and 100% positive cells were seen for certain antigens (Enblad *et al.*, unpublished observation). Furthermore, the heterogeneity may complicate the use of monoclonal antibodies in tumour imaging and therapy. Thus, monoclonal immunotherapy devices may be more efficient in antigenically less heterogeneous cancers. However, immunohistochemical screening may help to identify potentially the most appropriate antibodies for *in vivo* applications, as e.g. anti-CEA in the present study, which had a broad spectrum of reactivity both in primary tumours and metastasis.

An immunohistochemical classification based upon samples much larger than preoperative biopsies seems to be more accurate (Table III). The staining patterns did not correlate with degree of differentiation and Dukes' stage (Table II), which so far gives the best prediction of clinical course. However, the prognostic relevance may not be definitely assessed until the patients have been followed for longer time periods and the immunohistochemical profiles related to patient survival.

The authors wish to thank Mrs Stella Bonifacino for excellent technical assistance. The study was supported by grants from the Swedish Cancer Society (project No. 1921-B85-02XA), from the Swedish Society of Medical Sciences, and from the Medical Council of the Swedish Life Insurance Companies.

### References

ARENDS, J.W., WIGGERS, T., VERSTIJNEN, C., HILGERS, J. & BOSMAN, F.T (1984*a*). Gastrointestinal cancer-associated antigen (GICA) immunoreactivity in colorectal carcinoma in relation to patient survival. *Int. J. Cancer*, **34**, 193.

ARENDS, J.W., WIGGERS, T., THIJS, C.T., VERSTIJEN, C., SWAEN, G.J.V. & BOSMAN, F.T. (1984*b*). The value of secretory component (SC) immunoreactivity in diagnosis and prognosis of colorectal carcinomas. *Am. J. Clin. Pathol.*, **82**, 267.

ATKINSON, B.F., ERNST, C.S., HERLYN, M., STEPLEWSKI, Z., SEARS, H.F. & KOPROWSKI, H. (1982). Gastrointestinal cancer-associated antigen in immunoperoxidase assay. *Cancer Res.*, **42**, 4820.

BRATTAIN, M.G., PRETLOW, T.P. & PRETLOW, T.G. (1977). Cell fraction of large bowel cancer. *Cancer*, **40**, 2479.

BRATTAIN, M.G., GREEN, C., KIMBALL, P.M., MARKS, M. & KHALED, M. (1979). Iseoenzymes of B-hexosaminidase from normal rat colon and colonic carcinoma. *Cancer Res.*, **39**, 4083.

BRATTAIN, M.G., FINE, W.D., KHALED, F.W., THOMPSON, J. & BRATTAIN, D.E. (1981). Heterogeneity of malignant cells from a human colonic carcinoma. *Cancer Res.*, **41**, 1751.

CHEN, H.T. & KABAT, E.A. (1985). Immunochemical studies on blood groups. The combining site specificities of mouse monoclonal hybridoma anti-A and anti-B. *J. Biol. Chem.*, **260**, 13208.

CHEN, T.R., HAY, R.J. & MACY, M.L. (1982). Karyotype consistency in human colorectal carcinoma cell lines established *in vitro*. *Cancer Gen. Cytogen.*, **6**, 93.

COOPER, H.S., COX, J. & PATCHEFSKY, A.S. (1978). Immunohistologic study of blood group substances in polyps of the distal colon. *Am. J. Clin. Pathol.*, **73**, 345.

COOPER, H.S. & HAESSLER, W.E. (1978). Blood group substances as tumour antigens in the distal colon. *Am. J. Clin. Pathol.*, **69**, 594.

CUMMINGS, B.J. (1984). Adjuvant radiation therapy for rectal adenocarcinoma. *Dis. Colon Rectum*, **27**, 826.

DAAR, A.S. & FABRE, J.W. (1983). The membrane antigens of human colorectal cancer cells: Demonstration with monoclonal antibodies of heterogeneity within and between tumours and of anomalous expression of HLA-DR. *Eur. J. Cancer Clin. Oncol.*, **19**, 209.

DENK, H., TAPPEINER, G., DAVIDOVITS, A., ECKERSTORFER, R. & HOLZNER, J.H. (1974*a*). Carcinoembryonic antigens and blood group substances in carcinomas of the stomach and colon. *J. Natl Cancer Inst.*, **53**, 933.

DENK, H., TAPPEINER, G. & HOLZNER, J.H. (1974*b*). Blood group substances (BG) as carcinofetal antigens in carcinomas of the distal colon. *Eur. J. Cancer*, **10**, 487.

DENK, H., HOLZNER, J.H. & OBIDITSCH-MAYER, I. (1975). Epithelial blood group antigens in colon polyps. I. Morphologic distribution and relationship to differentiation. *J. Natl Cancer Inst.*, **54**, 1313.

DEXTER, D.L., BARBOSA, J.A. & CALABRESI, P. (1979). N, N-Dimethyl-formamide-induced alteration of culture characteristics and loss of tumorigenicity in cultures of human colon carcinoma cells. *Cancer Res.*, **39**, 1020.

EDWARDS, P.A.W. (1985). Heterogeneous expression of cell-surface antigens in normal epithelia and their tumours, revealed by monoclonal antibodies. *Br. J. Cancer*, **51**, 149.

ENBLAD, P., GLIMELIUS, B., BENGTSSON, A., PONTÉN, J. & PÅHLMAN, L. (1985). DNA content in carcinoma of the rectum and rectosigmoid. *Acta path, microbiol. immunol. scand.* Sect. A, **93**, 277.

ENBLAD, P., GLIMELIUS, B., BUSCH, C. & 4 others (1986). Comparative immunohistochemical demonstration of difucosylated carbohydrate antigens and CEA in adenomas and carcinomas of the rectum and rectosigmoid. *Anticancer Res.*, **6**, 139.

ERNST, C., ATKINSON, B., WYSOCKA, M. & 5 others (1984). Monoclonal antibody localization of Lewis antigens in fixed tissue. *Lab. Invest.*, **50**, 394.

FIDLER, I.J. (1978). Tumor heterogeneity and the biology of cancer invasion and metastasis. *Cancer Res.*, **38**, 2651.

HAKOMORI, S. (1980). Possible role of glycolipid in development, cell growth regulation and transformation. *Tumor Cell Surf. Malign.*, 873.

HAMMARSTRÖM, S., ENGVALL, E. & FELD, S. (1977). Quantitation of CEA by enzyme-linked immunoadsorbent assay (ELISA) and specificity studies. In *Tumour Markers*, Sixth Tenovus Workshop, p. 252 (eds.) Griffiths, *et al.*, Alpha Omega Publishing Ltd: Cardiff, UK.

KIMBALL, P.M. & BRATTAIN, M.G. (1980). Isolation of a cellular subpopulation from a human colonic carcinoma cell line. *Cancer Res.*, **40**, 1574.

KIMBALL, P.M., BRATTAIN, M.G. & PITTS, A.M. (1978). A soft-agar procedure for measuring growth of human colonic carcinomas. *Br. J. Cancer*, **37**, 1015.

LIMAS, C., LANGE, P., FRALEY, E.E. & VESSELLA, R.L. (1979). A, B, H antigens in transitional cell tumours of the urinary bladder. *Cancer*, **44**, 2099.

LINDHOLM, L., HOLMGREN, J., SVENNERHOLM, L. & 5 others (1983). Monoclonal antibodies against gastrointestinal tumour associated antigens isolated as monosialogangliosides. *Int. Arch Allergy Appl. Immun.*, **71**, 178.

MAGNANI, J.L., BROCKHAUS, M., SMITH, D.F. & 5 others (1981). A monosialoganglioside is a monoclonal antibody-defined antigen of colon carcinoma. *Science*, **212**, 55.

McCORMACK, S.A. (1984). Mixed cell populations in human mammary cancer. *Rev. Endocrine-Related Cancer*, **17**, 17.

NILSSON, O., MÅNSSON, J.-E., LINDHOLM, L. & 2 others (1985). Sialosyllactotetraosylceramide, a novel ganglioside antigen detected in human carcinomas by a monoclonal antibody. *FEBS Lett.*, **182**, 398.

NOWELL, P.C. (1976). The clonal evolution of tumor cell populations. *Science*, **194**, 23.

OLSSON, L., SÖRENSEN, H.R. & BEHNKE, O. (1984). Intratumoral phenotypic diversity of cloned human lung tumor cell lines and consequences for analyses with monoclonal antibodies. *Cancer*, **54**, 1757.

PETERSEN, S.E., BICHEL, P. & LORENTZON, M. (1979*a*). Flow cytometric demonstration of tumour cell subpopulations with different DNA content in human colo-rectal carcinoma. *Eur. J. Cancer*, **15**, 383.

PETERSEN, S.E., LORENTZON, M. & BICHEL, P. (1979*b*). A mosaic subpopulation structure of human colorectal carcinoma demonstrated by flow cytometry. In *Flow cytometry IV*, p. 412 (eds.) Laerum *et al.*, Universitetsförlaget: Bergen-Oslo-Tromsö, Norway.

PRETLOW, T.P., GLOVER, G.L. & PRETLOW, T.G. (1977). Purification of malignant cells and lymphocytes from a rat transplantable mucinous adenocarcinoma of the colon by isokinetic sedimentation in gradients of Ficoll. *J. Natl Cancer Inst.*, **59**, 981.

PÅHLMAN, L., GLIMELIUS, B. & GRAFFMAN, S. (1985). Pre-versus postoperative radiotherapy in rectal carcinoma: an interim report from a randomized multicentre trial. *Br. J. Surg.*, **72**, 961.

ROGNUM, T.O., BRANDTZAEG, P. & THORUD, E. (1983). Is heterogeneous expression of HLA-DR antigens and CEA along with DNA-profile variations evidence of phenotypic instability and clonal proliferation in human large bowel carcinomas? *Br. J. Cancer*, **48**, 543.

SZULMAN, A.E. (1962). The histological distribution of blood group substances in man as disclosed by immunofluorescence. II. The H antigen and its relation to A and B antigens. *J. Exp. Med.*, **115**, 977.

SZULMAN, A.E. & MARCUS, D.M. (1973). The histologic distribution of the blood group substances in man as disclosed by immunofluorescence. VI. The Le a and Le b antigens during fetal development. *Lab. Invest.*, **28**, 565.

WEINSTEIN, R.S., COON, J., ALROY, J. & DAVIDSOHN, I. (1981). Tissue-associated blood group antigens in human tumors. In: *Diagnostic Immunohistochemistry*, p. 239. (ed.) DeLellis, Masson: New York.

WIGGERS, T., ARENDS, J.W., VERSTIJNEN, C., MOERKERK, P.M. & BOSMAN, F.T. (1986). Prognostic significance of CEA immunoreactivity patterns in large bowel carcinoma tissue. *Br. J. Cancer*, **54**, 409.

WILEY, E.L., MENDELSOHN, G. & EGGLESTON, J.C. (1981). Distribution of carcinoembryonic antigens and blood group substances in adenocarcinoma of the colon. *Lab. Invest.*, **44**, 507.